# Differential Expression of Superoxide Dismutase Genes in Aphid-Stressed Maize (*Zea mays* L.) Seedlings

**DOI:** 10.1371/journal.pone.0094847

**Published:** 2014-04-10

**Authors:** Hubert Sytykiewicz

**Affiliations:** Siedlce University of Natural Sciences and Humanities, Department of Biochemistry and Molecular Biology, Siedlce, Poland; University of South Florida College of Medicine, United States of America

## Abstract

The aim of this study was to compare the expression patterns of superoxide dismutase genes (*sod2*, *sod3.4*, *sod9* and *sodB*) in seedling leaves of the *Zea mays* L. Tasty Sweet (susceptible) and Ambrozja (relatively resistant) cultivars infested with one of two hemipteran species, namely monophagous *Sitobion avenae* F. (grain aphid) or oligophagous *Rhopalosiphum padi* L. (bird cherry-oat aphid). Secondarily, aphid-elicited alternations in the antioxidative capacity towards DPPH (1,1-diphenyl-2-picrylhydrazyl) radical in insect-stressed plants were evaluated. Comprehensive comparison of expression profiles of the four *sod* genes showed that both insect species evoked significant upregulation of three genes *sod2*, *sod3.4* and *sod9*). However, aphid infestation affected non-significant fluctuations in expression of *sodB* gene in seedlings of both maize genotypes. The highest levels of transcript accumulation occurred at 8 h (*sod2* and *sod3.4*) or 24 h (*sod9*) post-infestation, and aphid-induced changes in the expression of *sod* genes were more dramatic in the Ambrozja cultivar than in the Tasty Sweet variety. Furthermore, bird cherry-oat aphid colonization had a more substantial impact on levels of DPPH radical scavenging activity in infested host seedlings than grain aphid colonization. Additionally, Ambrozja plants infested by either hemipteran species showed markedly lower antioxidative capacity compared with attacked Tasty Sweet plants.

## Introduction

The global production and economic importance of maize (*Zea mays* L.) have steadily increased during the last decade. This increment is likely due to increased worldwide distribution, the adaptability of maize to multifarious environmental conditions, and the introduction of high-yield varieties [1]–[2]. Maize is a substantial source of raw materials for the pulp and paper industries as well as for fermentation processes in biogas and bioethanol synthesis [1]–[4]. Additionally, *Z. mays* is an important model organism in plant experimental biology, such as studies of the molecular basis of plant-insect interactions, pest resistance mechanisms, and genetic, biochemical and physiological aspects of plant development [1]–[3].

Aphids (Hemiptera, Aphidoidea) are one of the most destructive groups of insects colonizing a large number of maize varieties [5]–[8]. Infestation of the host plants by these piercing-sucking hemipterans lead to a wide spectrum of deleterious effects, including ultrastructural organ damage, severe depletion of phloem sap constituents, and perturbation of many fundamental physiological processes such as photosynthesis, cellular respiration, growth, and development. Long-term and/or large-scale aphid colonization may result in additional detrimental effects such as large chlorotic lesions, stress-induced premature senescence, apoptosis, and local necrosis [9]–[17]. Furthermore, these arthropods serve as vectors for a broad range of plant-pathogenic viruses [18]–[19]. Aphid watery saliva, which is injected into target plant tissues, contains a broad collection of hydrolytic enzymes, metabolic effectors, and toxic compounds that may also stimulate the host to excessive formation of reactive oxygen species (ROS) [20]–[24]. Circumstantial disturbance of intracellular redox homeostasis in stressed plants may result in diverse cytotoxic effects and initiate the cascade of reactions leading to programmed cell death. Prolonged overproduction of various ROS can lead to peroxidation of membrane lipids and pigments, denaturation of proteins, damage to DNA, and fragmentation of polysaccharides [25]–[29].

Higher plants have evolved a complex network of antioxidant systems to counteract elevated ROS levels produced in response to unfavorable environmental conditions. This sophisticated machinery encompasses a wide range of lipid- and water-soluble antioxidants (e.g., tocopherols, *β*-carotene, ubiquinone, ascorbate, glutathione) and antioxidant enzymes such as superoxide dismutases (SODs), catalase, glutathione transferase, glutathione peroxidase, and ascorbate peroxidase [25], [27], [30]–[36]. SODs are a group of metalloenzymes that constitute the primary line of antioxidative defense by catalyzing the dismutation reaction of superoxide anion radical (O_2_^–^) to oxygen (O_2_) and hydrogen peroxide (H_2_O_2_). In plants, three types of SODs (Cu/ZnSODs, FeSODs and MnSODs) have been identified, differing in the metal cofactor present within the active site. Cu/ZnSOD isoforms are found in the cytosol, chloroplasts, mitochondria, peroxisomes, and extracellular space, FeSOD isozymes are present in chloroplasts, and MnSODs are localized to mitochondria [25], [37]–[42]. Significant modulations in SOD activity have been observed in a variety of plants exposed to a broad range of environmental stresses, such as drought [43]–[47], high or low temperature [48]–[50], ultraviolet-B irradiation [51]–[52], darkness [53], high salinity [54], nitrogen deficiency [55], supplementation with carbohydrates [56], herbicide treatment [57], heavy metal exposure [28], [58]–[60], magnetic field influence [61], and pathogen infection [62]–[66].

Although aphid-stimulated physiological and biochemical modulations of a wide spectrum of infested host systems have been extensively studied, the specific effects on the antioxidant machinery remain unclear. To date, there are no available studies regarding the regulation of *sod* genes and antiradical activity against DPPH• in aphid-susceptible and aphid-resistant maize varieties. There is a lack of comparative data regarding the influence of mono- and oligophagous aphid species on the expression patterns of *sod* genes and levels of the antioxidative capacity in colonized *Z. mays* seedlings. It has been hypothesized that susceptible and resistant maize cultivars differ in their transcriptional regulation of *sod* genes and antioxidant activity in response to aphid infestation. Hence, the main purpose of the study was to compare the transcriptional activity of *sod* genes (*sod2*, *sod3.4*, *sod9* and *sodB*) in seedling leaves of the maize Tasty Sweet (susceptible) and Ambrozja (relatively resistant) cultivars colonized by monophagous *Sitobion avenae* F. (grain aphid) or oligophagous *Rhopalosiphum padi* L. (bird cherry-oat aphid). Additionally, it was evaluated whether changes in the relative expression of these *sod* genes and DPPH radical scavenging activity in the stressed maize plants reflect levels of aphid infestation.

## Methods

### Plant material

Tasty Sweet and Ambrozja *Z. mays* seeds were purchased from local garden supply companies. Plants were grown in a climate controlled chamber at 22±2°C/16±2°C (day/night), relative humidity of 65±5%, light intensity 100 μM m^−2^ s^−1^, and a long-day photoperiod (light 16 h: dark 8 h). Seedlings were separately planted in round plastic pots (10×9 cm; diameter × height) filled with general-purpose horticultural substrate with no additional fertilization. According to Sytykiewicz et al., Tasty Sweet and Ambrozja maize varieties were classified as aphid-susceptible and aphid-relatively resistant, respectively [8].

### Aphids

Apterous parthenogenetic females of the two aphid species were gathered from cereal plants in the Siedlce district (Poland) and reared for 1 year on the seedlings of *Triticum aestivum* L. (Tonacja variety) at the Department of Biochemistry and Molecular Biology, University of Natural Sciences and Humanities, Siedlce. The insects were maintained in the climate controlled plant growth chamber described above. To sustain the aphid populations, new *T. aestivum* plants were added every week, and the old plants were removed after the aphids had settled on the new seedlings. Adult wingless aphids were used in the experiments.

### Experimental design

The bioassays were performed on leaves of 14-day-old seedlings of the two maize cultivars that were artificially infested with 10, 20, 40, or 60 adult apterous females aphids per plant. Control plants were not infested with the insects. Transcriptional activity of the SOD isozyme genes (*sod2*, *sod3.4*, *sod9* and *sodB*) and the antioxidative capacity in the leaves of maize seedlings were measured at 1, 2, 4, 8, 24, 48, and 72 h post-initial aphid infestation (hpi). Aphid-stressed and control *Z. mays* seedlings were caged individually in transparent plastic cylinders (20×50 cm; diameter × height) covered with nylon mesh. To terminate each series of experiments, aphids were removed from the infested maize plants, and the leaves were excised and immediately subjected to further analysis.

### Assay of DPPH radical scavenging activity

The antioxidative activity of maize extracts towards DPPH• (1,1-diphenyl-2-picrylhydrazyl radical) was determined according to the method of Brand-Williams et al. [67], with minor modifications. Freshly harvested seedling leaves (1.0 g) of *Z. mays* were homogenized with 15 cm^3^ of methanol, and next, the samples were vigorously vortexed for 30 min. The cell-free homogenates were filtered through four layers of mesh gauze and centrifuged at 10,000 *g* for 15 min, and after that the pellet was discarded. The reaction mixture consisted of 20 mm^3^ of the supernatant and 365 mm^3^ of DPPH methanolic solution (0.04%; *w/v*), the control probe and the samples were incubated at room temperature for 30 min. L-ascorbic acid was used as a positive control. The absorbance at 517 nm was measured using an Epoch UV-Vis microplate spectrophotometer (BioTek, USA). The antioxidative activity of the maize extracts was calculated using the following formula: DPPH• scavenging activity (%)  =  A_0_ – A_S_/A_0_×100 (where A_0_ is absorbance of the control - DPPH• solution; A_S_ – absorbance of the tested *Z. mays* samples).

### Total RNA isolation and quantification

Total RNA was extracted from the aphid-colonized and control *Z. mays* seedlings. The leaves were harvested and immediately homogenized in liquid nitrogen using a sterile, RNase-free ceramic mortar and pestle. Total RNA was isolated using the Spectrum Plant Total RNA kit (Sigma Aldrich, Poland), and residual genomic DNA was enzymatically hydrolyzed with the On-Column DNase I Digestion Set (Sigma Aldrich, Poland). The purified RNA was quantified using the Epoch UV-Vis microplate spectrophotometer. Additionally, A_260/280_ and A_260/230_ ratios were calculated to estimate RNA integrity and purity. Only RNA samples of high quality (A_260/280_>2.0 and A_260/230_>1.8) were selected for further analysis.

### cDNA synthesis

Purified total RNA (1 μg) was used for reverse transcription using the RevertAid Premium First Strand cDNA Synthesis kit (Fermentas, Poland) and oligo(dT)_18_ primers. Two types of negative control reactions were prepared: no template and no reverse transcription.

### Quantitative PCR

Transcriptional activity of *sod* genes in the leaves of aphid-infested and control *Z. mays* seedlings was analyzed by quantitative PCR. Expression levels of *sod2*, *sod3.4*, and *sodB* were estimated using gene-specific TaqMan Gene Expression assays (Life Technologies, Poland). Table S1 lists the identification numbers of the assays and reference sequences. The gene encoding glyceraldehyde 3-phosphate dehydrogenase (*gapdh*) was used an the internal control. The expression of *gapdh* and *sod9* genes was quantified with Custom TaqMan Gene Expression assays. Primer sequences and fluorescent probe are shown in Table S2. Expression of the target genes was evaluated in 96-well microplates on the StepOne Plus Real-Time PCR System using the StepOnePlus Software v2.3 (Applied Biosystems, USA). The final PCR reaction volume was 20 mm^3^ and consisted of 10 mm^3^ 2× TaqMan Fast Universal PCR Master Mix, 1 mm^3^ 20× TaqMan Gene Expression assay mixture (containing a pair of primers and a TaqMan 6-carboxyfluorescein-labeled minor groove binder probe), 4 mm^3^ of cDNA, and 5 mm^3^ of RNase-free water. Amplification curves were generated under the following thermal parameters: 95°C for 20 s (activation of AmpliTaq Gold DNA Polymerase (Life Technologies, Poland), followed by 40 cycles of 95°C for 1 s and 60°C for 20 s. The comparative C_T_ (ΔΔC_T_) method of Livak and Schmittgen [68] was used to calculate the relative expression of each target gene, and the mean values are presented as the fold change ± standard deviation (SD) in the transcript in aphid-infested samples compared with the controls.

### Statistical analysis

All data are expressed as the mean ± SD of three independent biological replicates. Significance of differences in the expression of *sod* genes and levels of the antioxidative capacity between the aphid-colonized seedlings of each variety and the relevant control plants was assessed by a factorial analysis of variance (ANOVA). The factorial ANOVA comprised the evaluation of four factors: maize genotype (Ambrozja and Tasty Sweet), aphid species (*R. padi* and *S. avenae*), treatment (10, 20, 40 and 60 aphids per plant), and time post-infestation (0, 1, 2, 4, 8, 24, 48 and 72 hpi). Subsequently, the *post-hoc* analysis was carried out by using the Tukey's test (P-values ≤0.05 were considered statistically significant). The obtained results were analyzed using STATISTICA 10 software (StatSoft, Poland).

## Results

### The influence of aphid colonization on the antioxidative potential of maize seedlings

The performed studies revealed that the investigated aphid species (*R. padi* or *S. avenae*) led to a decrement in levels of the antioxidative capacity towards DPPH radical within the seedling leaves of Ambrozja and Tasty Sweet genotypes in relation to the uninfested plants (Table 1, 2). More severe decline in DPPH radical scavenging activity in the insect-colonized maize seedlings was influenced by the bird cherry-oat aphid feeding when compared to *S. avenae* infestation (e.g. 72-hour feeding of 60 *R. padi* insects evoked 17% and 2% greater decrease in the analysed parameter in Ambrozja and Tasty Sweet plants, respectively, when compared to changes stimulated by the grain aphid). Additionally, seedlings of Ambrozja genotype colonized by the tested hemipteran species characterized by more significant deceleration in levels of the antioxidative potential (e.g. 1–5% and 2–19% greater decrease in plants infested by 10 and 60 aphids, respectively) in comparison with Tasty Sweet variety. Conducted experiments demonstrated that the scale of aphid-triggered depletion in the antioxidant activity in maize seedlings was dependent on duration of aphid exposure and insect density per plant. Exemplarily, the highest infestation level (60 *R. padi* aphids per plant) and the longest duration of insect colonization (72 hpi) resulted in 33 and 14% decrease of the analysed parameter in Ambrozja and Tasty Sweet plants, accordingly, while a lower decline (16 and 12%, respectively) was estimated in the investigated maize cultivars infested by the grain aphid. On the other hand, time-course analysis revealed that the antioxidative capacity in Tasty Sweet seedlings infested by 10 *S. avenae* aphids remained unchanged at different intervals of aphid exposure (1–72 hpi) when compared to the relevant controls, whereas colonization of these plants by the same number of bird cherry-oat aphid individuals led to a slight decline (3–4%) at 48 and 72 hpi, respectively. Ambrozja plants infested by the lowest number of *R. padi* or *S. avenae* aphids (10 per plant) responded a similar decrement (2–9% or 2–6%, respectively) in levels of the antioxidative potential after 8–24 hours of aphid feeding. The factorial analysis of variance evidenced that four tested factors and interactions between these parameters statistically affected levels of the antioxidative capacity in the maize seedlings (Table 3).

**Table 1:** DPPH radical scavenging activity (%) of methanolic extracts prepared from *R. padi*-infested maize seedlings.

Duration of aphid colonization (hpi)	Levels of infestation (number of aphids per seedling)				
	0	10	20	40	60
**Ambrozja cultivar**					
0	28.2±1.5a	28.2±1.5a	28.2±1.5a	28.2±1.5a	28.2±1.5a
1	28.4±1.6a	28.1±1.4a	28.6±1.7a	28.3±1.5a	27.9±1.4a
2	28.1±1.4a	28.3±1.5a	28.2±1.5a	27.6±1.4a	26.7±1.3ab
4	28.6±1.7a	28.4±1.6a	28.6±1.5a	26.9±1.3b	25.3±1.1b
8	28.8±1.9a	28.8±1.7a	27.7±1.4a	25.2±1.0b	22.4±0.8c
24	29.0±2.1a	27.3±1.4ab	26.8±1.3b	24.0±0.8bc	20.2±0.7c
48	28.3±1.6a	26.8±1.3b	25.2±1.1b	21.4±0.7c	16.3±0.5d
72	28.5±1.7a	25.7±1.1b	24.5±1.0b	18.6±0.6c	12.6±0.3d
**Tasty Sweet cultivar**					
0	25.6±1.3a	25.6±1.3a	25.6±1.3a	25.6±1.3a	25.6±1.3a
1	25.2±1.2a	25.4±1.2a	25.3±1.2a	25.1±1.1a	25.2±1.2a
2	25.5±1.3a	25.8±1.4a	25.2±1.2a	25.6±1.3a	25.1±1.1a
4	25.3±1.2a	25.1±1.1a	25.6±1.3a	24.9±1.0a	24.6±0.9ab
8	24.9±1.0a	25.0±1.0a	25.1±1.1a	24.7±0.9ab	24.5±0.9ab
24	25.0±1.0a	25.5±1.2a	24.8±1.0a	24.1±0.9ab	23.2±0.8b
48	25.1±1.1a	24.6±1.0a	24.6±0.9a	23.5±0.8b	22.4±0.7b
72	25.7±1.3a	24.7±0.9a	24.0±0.9ab	22.9±0.7b	20.8±0.5bc

Values are presented as the mean ± SD of three replicates; hpi-hours post-initial aphid infestation. Antioxidant capacity of the maize extracts is expressed as the percent inhibition of DPPH radical. The average values in rows denoted by different letters are statistically significant (Tukey's test; P≤0.05).

**Table 2:** DPPH radical scavenging activity (%) of methanolic extracts prepared from *S. avenae*-infested maize seedlings.

Duration of aphid colonization (hpi)	Levels of infestation (number of aphids per seedling)				
	0	10	20	40	60
**Ambrozja cultivar**					
0	28.2±1.5a	28.2±1.5a	28.2±1.5a	28.2±1.5a	28.2±1.5a
1	28.4±1.6a	28.1±1.4a	28.5±1.7a	28.6±1.7a	28.3±1.6a
2	28.1±1.4a	28.9±2.0a	28.1±1.4a	28.7±1.8a	28.1±1.4a
4	28.6±1.7a	28.2±1.5a	28.7±1.8a	27.5±1.4a	26.9±1.3b
8	28.8±1.9a	28.3±1.6a	28.4±1.6a	27.3±1.4ab	26.5±1.3b
24	29.0±2.1a	28.2±1.5a	28.0±1.4a	26.4±1.3b	26.1±1.2b
48	28.3±1.6a	27.5±1.4a	27.2±1.4ab	24.9±1.0bc	25.0±1.0bc
72	28.5±1.7a	27.0±1.3a	26.8±1.3ab	24.8±1.0ab	23.9±0.8c
**Tasty Sweet cultivar**					
0	25.6±1.3a	25.6±1.3a	25.6±1.3a	25.6±1.3a	25.6±1.3a
1	25.2±1.2a	25.2±1.2a	25.4±1.2a	25.8±1.3a	25.0±1.1a
2	25.5±1.3a	25.7±1.3a	25.6±1.3a	25.9±1.4a	25.2±1.2a
4	25.3±1.2a	25.2±1.2a	25.0±1.1a	25.4±1.2a	24.8±1.0a
8	24.9±1.0a	25.1±1.1a	24.9±1.0a	24.8±1.1a	24.2±0.9ab
24	25.0±1.0a	25.5±1.3a	24.7±1.0a	24.6±0.9ab	23.3±0.8b
48	25.1±1.1a	25.3±1.2a	24.4±0.9ab	24.3±0.9ab	22.8±0.7b
72	25.7±1.3a	25.2±1.2a	24.2±0.9ab	23.6±0.8b	22.6±0.7bc

Values are presented as the mean ± SD of three replicates; hpi - hours post-initial aphid infestation. Antioxidant capacity of the maize extracts is expressed as the percent inhibition of DPPH radical. The average values in rows denoted by different letters are statistically significant (Tukey's test; P≤0.05).

**Table 3:** Results of the factorial ANOVA of experimental factors (maize genotype, aphid species, treatment = infestation level, time post-infestation) and their interactions influencing the antioxidative activity in maize seedlings.

Parameter	Df	*F*-value	*P*-value
Maize genotype (M)	1	628.45	≤0.001
Aphid species (A)	2	97.6	≤0.001
Treatment (T)	3	45.6	≤0.001
Time post-infestation (TPI)	7	44.7	≤0.001
A × M	2	79.2	≤0.001
A × T	6	18.5	≤0.001
M × T	3	17.1	≤0.001
A × TPI	14	16.7	≤0.001
M × TPI	7	9.2	≤0.001
T × TPI	21	5.4	≤0.001
A × M × T	6	11.0	≤0.001
A × M × TPI	14	9.2	≤0.001
A × T × TPI	42	10.2	≤0.001
M × T × TPI	21	8.1	0.009
A × M × T × TPI	42	5.7	0.035

Df, degree of freedom; values of P≤0.05 were considered statistically significant.

### Expression profiles of *sod2* in *R. padi*– and *S. avenae*–stressed maize seedlings

**Figure 1:**
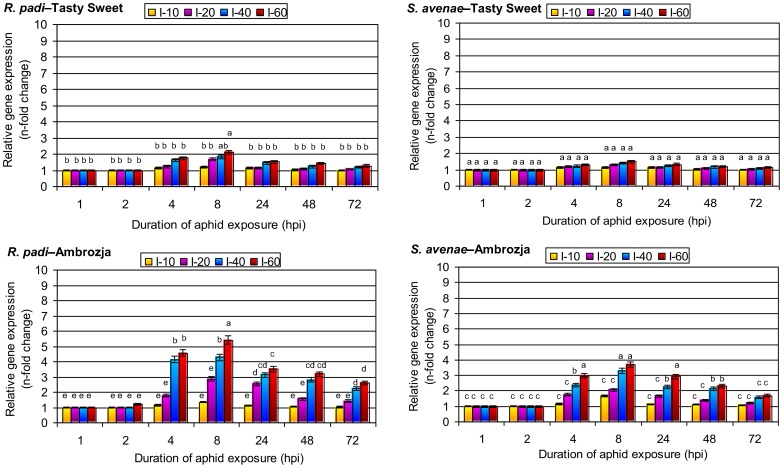
Transcription of *sod2* in aphid-colonized seedlings of Tasty Sweet (susceptible) and Ambrozja (relatively resistant) maize varieties. (I) – infestation level (number of aphids per seedling). Values represent the average fold change in relative gene expression (± SD) in the aphid-infested maize seedlings compared with control (uninfested) plants. For each experimental combination, three independent biological replicates were performed. Different letters indicate statistically significant differences (Tukey's test; P≤0.05).

Short-term (1 or 2 hpi) feeding by the aphids did not alter *sod2* expression, except in the case of Ambrozja plants infested with 60 *R. padi* at 2 hpi in which there was a 20% increase in transcript levels compared with the control (Figure 1). After 4 h of aphid feeding, the *sod2* transcript level increased in both cultivars with both aphid species, ranging from a 1.3-fold increase in Tasty Sweet seedlings infested with 60 *S. avenae* to a 4.6-fold increase in Ambrozja plants infested with 60 *R. padi*. Maximal enhancement of gene expression in aphid-infested seedling leaves in both *Z. mays* cultivars was seen at 8 hpi with 60 aphids per plant (1.5- to 5.4-fold elevation). However, the insect-triggered increase in *sod2* expression was greater in Ambrozja (5.4- and 3.7-fold upregulation by *R. padi* and *S. avenae*, respectively) than in Tasty Sweet plants (2.1- and 1.5-fold upregulation, respectively). The enhanced *sod2* expression in both maize genotypes became less pronounced with prolonged insect colonization (1.3- to 3.5-fold higher expression at 24 hpi, and 1.2- to 3.2-fold increase at 48 hpi, depending on the aphid species and maize cultivar). Consequently, the smallest difference in *sod2* expression between aphid-infested and control plants was seen after long-term aphid infestation (72 hpi). For example, at 72 hpi, 60 *R. padi* aphids stimulated a 28% increase in *sod2* expression in Tasty Sweet plants and a 160% increase in Ambrozja plants, whereas the same number of *S. avenae* aphids increased *sod2* expression by 70% in Tasty Sweet and 15% in Ambrozja plants. The factorial ANOVA testing proved that four experimental parameters and their interactions significantly influenced the transcriptional activity of *sod3.4* gene in *Z. mays* plants (Table 4).

**Table 4:** Results of the factorial ANOVA of experimental factors (maize genotype, aphid species, treatment = infestation level, time post-infestation) and their interactions influencing the relative expression of *sod2* and *sod3.4* genes in maize seedlings.

Parameter	Df	*F*-value	*P*-value	*F*-value	*P*-value
		*sod2* gene		*sod3.4* gene	
Maize genotype (M)	1	48.4	≤0.001	91.8	≤0.001
Aphid species (A)	2	83.9	≤0.001	129.7	≤0.001
Treatment (T)	3	55.0	≤0.001	69.7	≤0.001
Time post-infestation (TPI)	7	33.1	≤0.001	51.4	≤0.001
A × M	2	18.5	≤0.001	28.8	≤0.001
A × T	6	15.0	≤0.001	25.1	≤0.001
M × T	3	7.1	≤0.001	13.8	≤0.001
A × TPI	14	9.6	≤0.001	15.5	≤0.001
M × TPI	7	7.9	≤0.001	21.1	≤0.001
T × TPI	21	5.4	≤0.001	8.4	≤0.001
A × M × T	6	3.5	≤0.001	4.4	≤0.001
A × M × TPI	14	3.7	≤0.001	7.5	≤0.001
A × T × TPI	42	2.6	≤0.001	3.0	≤0.001
M × T × TPI	21	1.9	0.010	5.2	≤0.001
A × M × T × TPI	42	1.8	0.002	2.6	≤0.001

Df, degree of freedom; values of P ≤ 0.05 were considered statistically significant.

### Effect of aphid infestation on the transcriptional activity of *sod3.4* in *Z. mays* tissues

**Figure 2:**
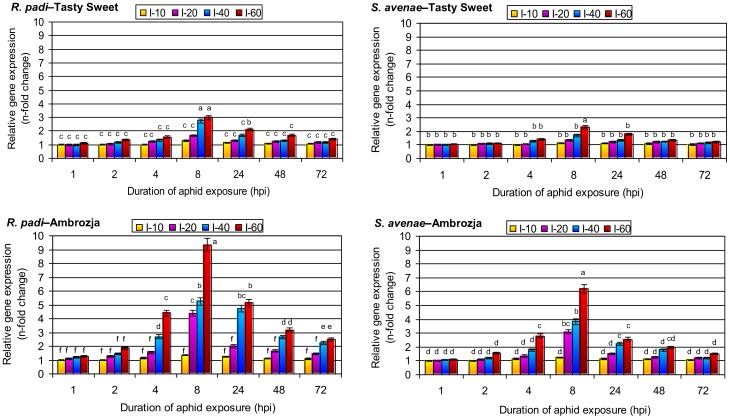
Transcription of *sod3.4* in aphid-colonized seedlings of the Tasty Sweet and Ambrozja maize varieties. (I) – infestation level (number of aphids per seedling). Values represent the average fold changes in relative gene expression (± SD) in the aphid-infested maize seedlings compared with control (non-infested) plants. For each experimental combination, three independent biological replicates were performed. Different letters indicate statistically significant differences (Tukey's test; P≤0.05).

Low and moderate aphid densities (10, 20 and 40 per plant) did not alter *sod3.4* expression at 1 hpi in Tasty Sweet seedlings. In contrast, 60 aphids per plant induced a 12% increase in *sod3.4* expression in *R. padi*–infested Tasty Sweet seedlings and a 5% increase in *S. avenae*–infested Tasty Sweet seedlings at 1 hpi (Figure 2). In the Ambrozja cultivar, the lowest density of either aphid species (10 per plant) did not alter *sod3.4* expression at 1 hpi, but all other aphid treatments led to elevated *sod3.4* expression relative to the control (ranging from a 12% increase in seedlings infested with 60 *S. avenae* per plant to an 86% increase in seedlings infested with 60 *S. avenae* per plant). Furthermore, at 4 hpi *sod3.4* transcript levels were higher in both maize cultivars than at the earlier time points, with the exception of Tasty Sweet seedlings infested with 10 aphids per seedling, for which no change in *sod3.4* expression was observed. The maximal increase in *sod3.4* expression in aphid-stressed maize plants occurred at 8 hpi with 60 aphids per plant (2.3- to 9.4-fold increase relative to control seedlings), and the increase was greater in Ambrozja (6.2- and 9.4-fold increase in *S. avenae*– and *R. padi*–infested seedlings, respectively) than in Tasty Sweet (2.3- and 3.0-fold increase in *S. avenae*– and *R. padi*–infested plants, respectively). Continued feeding of *R. padi* or *S. avenae* on the seedlings (24, 48 and 72 hpi) resulted in progressively lower *sod3.4* expression compared with 8 hpi. For example, 60 *R. padi* aphids per plant caused a 150% increase in *sod3.4* expression in Ambrozja seedlings and a 42% increase in expression in Tasty Sweet plants at 72 hpi. The same density of *S. avenae* resulted in less of an increase in expression compared with control plants (21 and 52% in Tasty Sweet and Ambrozja plants, respectively). The factorial ANOVA confirmed the significant effects of four tested parameters and their interactions on the relative *sod3.4* gene expression within maize seedlings (Table 4).

### Impact of *R. padi* or *S. avenae* colonization on *sod9* expression in maize plants

**Figure 3:**
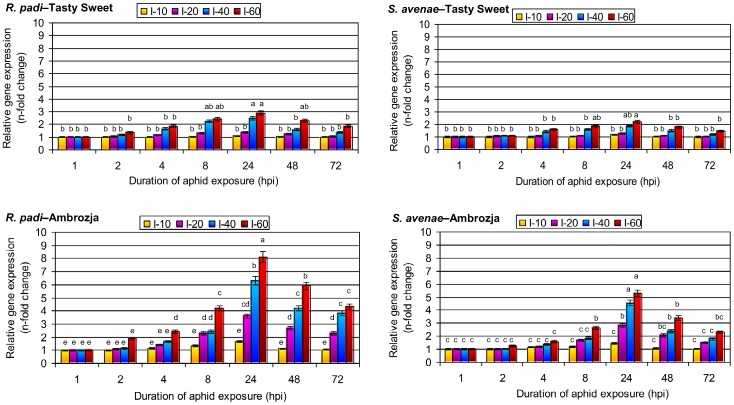
Transcription of *sod9* in aphid-colonized seedling leaves of the Tasty Sweet and Ambrozja maize varieties. (I) – infestation level (number of aphids per seedling). Values represent the average fold changes in relative gene expression (± SD) in the aphid-infested maize seedlings compared with control (non-infested) plants. For each experimental combination, three independent biological replicates were performed. Different letters indicate statistically significant differences (Tukey's test; P≤0.05).

At 1 hpi with bird cherry-oat or grain aphids, *sod9* expression in both cultivars did not differ from that measured in control plants (Figure 3). Additionally, colonization with only 10 of either insect species per plant did not alter *sod9* transcription at 2 hpi. Infestation of the maize plants with *R. padi* aphids at 20 per seedling evoked a slight increase in the relative *sod9* expression (6–10%), whereas more *R. padi* aphids (40 or 60 insects per seedling) resulted in a greater upregulation of *sod9*, ranging from a 10% increase in Tasty Sweet plants with 40 aphids per plant to a 90% increase in Ambrozja seedlings with 60 aphids per plant at 2 hpi relative to control plants. Prolonged aphid feeding (4 and 8 hpi) was associated with further augmentation in *sod9* transcription compared with the control, and the highest stimulation of *sod9* expression in the aphid-infested seedlings occurred at 24 hpi, with the Ambrozja cultivar showing a greater increase in the *sod9* transcript (1.4- to 5.3-fold and 1.7- to 8.1-fold increases in *S. avenae*– and *R. padi*–infested seedlings, respectively) than the Tasty Sweet variety (1.1- to 2.2-fold and 1.1- to 2.9-fold increases in *S. avenae*– and *R. padi*–infested plants, respectively). However, at 48 and 72 hpi the difference in transcriptional activity of *sod9* between the colonized and control maize seedlings was smaller than at 24 hpi. At 72 hpi with bird cherry-oat aphids, *sod9* expression was 4–85% higher in Tasty Sweet seedlings and 5–330% higher in Ambrozja seedlings than in controls. The increases in *sod9* expression were also dependent on aphid density, with 15–50% increase in expression at 40–60 *R. padi* aphids per Tasty Sweet seedling, and 80–130% increase (at 40–60 per seedling) in Ambrozja cultivar compared with control plants. However, lower numbers of insects colonizing the Tasty Sweet cultivar (10 *R. padi* and 10–20 *S. avenae* per plant) at 72 hpi did not alter *sod9* expression. The factorial analysis of variance revealed the statistically significant influence of four experimental parameters and their interactions on the amount of *sod9* transcript within the *Z. mays* plants (Table 5).

**Table 5:** Results of the factorial ANOVA of experimental factors (maize genotype, aphid species, treatment = infestation level, time post-infestation) and their interactions influencing the relative expression of *sod9* and *sodB* genes in maize seedlings.

Parameter	Df	*F*-value	*P*-value	*F*-value	*P*-value
		*sod9* gene		*sodB* gene	
Maize genotype (M)	1	1081.0	≤0.001	0.8	0.305
Aphid species (A)	2	1167.5	≤0.001	1.2	0.364
Treatment (T)	3	586.6	≤0.001	1.5	0.201
Time post-infestation (TPI)	7	485.4	≤0.001	0.9	0.508
A × M	2	363.3	≤0.001	1.3	0.275
A × T	6	188.3	≤0.001	1.0	0.416
M × T	3	118.1	≤0.001	0.9	0.434
A × TPI	14	145.3	≤0.001	0.7	0.721
M × TPI	7	191.6	≤0.001	0.6	0.743
T × TPI	21	70.0	≤0.001	1.1	0.367
A × M × T	6	44.0	≤0.001	1.2	0.279
A × M × TPI	14	59.5	≤0.001	1.0	0.401
A × T × TPI	42	21.7	≤0.001	0.8	0.748
M × T × TPI	21	24.9	≤0.001	0.7	0.760
A × M × T × TPI	42	8.7	≤0.001	1.1	0.315

Df, degree of freedom; values of P ≤ 0.05 were considered statistically significant.

### Aphid-evoked changes in *sodB* expression in leaf tissues of *Z. mays* seedlings

**Figure 4:**
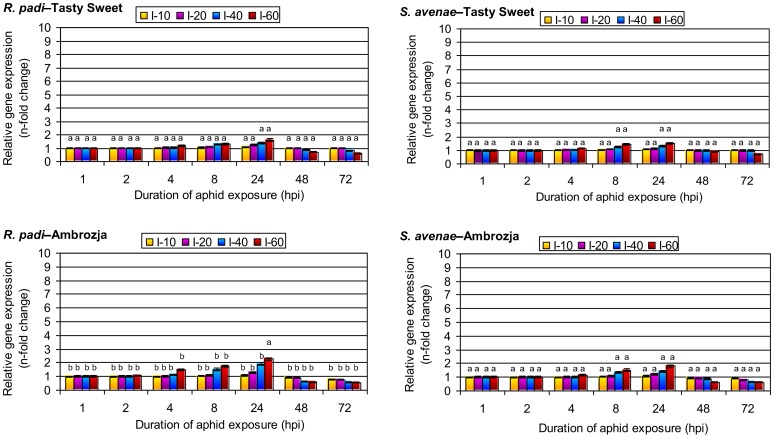
Transcription of *sodB* in aphid-colonized seedling leaves of the Tasty Sweet and Ambrozja maize varieties. (I) – infestation level (number of aphids per seedling). Values represent the average fold changes in relative gene expression (± SD) in the aphid-infested maize seedlings compared with control (non-infested) plants. For each experimental combination, three independent biological replicates were performed. Different letters indicate statistically significant differences (Tukey's test; P≤0.05).

Predation by both aphid species for 1 or 2 h did not affect *sodB* transcript levels in tissues of either *Z. mays* genotype (Figure 4). Similarly, infestation with only 10 *R. padi* or *S. avenae* per plant did not alter *sodB* expression up to 4 hpi. However, higher aphid densities (20, 40 and 60 per plant) slightly enhanced *sodB* expression relative to the control. For example, infestation with 60 *R. padi* per plant led to 17 and 49% increases in the *sodB* expression in Tasty Sweet and Ambrozja plants, respectively, whereas infestation with 60 *S. avenae* per plant led to 13 and 16% increases in Tasty Sweet and Ambrozja seedlings, respectively. After prolonged aphid infestation (8 hpi) similar increases in *sodB* expression were observed, ranging from a 5% increase in Tasty Sweet plants exposed to 10 *S. avenae* per plant to a 76% increase in Ambrozja seedlings exposed to 60 *R. padi* per plant. The largest increases in *sodB* expression occurred at 24 hpi, when 10–60 bird cherry-oat aphids per plant led to 10–62% and 12–127% increases in Tasty Sweet and Ambrozja plants, respectively, relative to the controls. *S. avenae* colonization (10–60 per plant) evoked slightly less of an increase in *sodB* expression, with 7–52% and 9–80% increases in Tasty Sweet and Ambrozja plants, respectively. Relative expression of *sodB* gene was downregulated at 48 and 72 hpi in Tasty Sweet seedlings infested with 40 or 60 *R. padi* per plant or 60 *S. avenae* per plant and in all infested Ambrozja plants, whereas *sodB* transcript amount was unchanged in Tasty Sweet seedlings infested with 10 or 20 *R. padi* per plant or 10, 20, or 40 *S. avenae* relative to control plants. The greatest suppression of *sodB* expression in tissues of both maize varieties relative to the controls occurred at 72 hpi. Amount of *sodB* transcript in the seedlings colonized with 60 *R. padi* per plant declined by 36 and 49% in the Tasty Sweet and Ambrozja plants, respectively, whereas *S. avenae* colonization reduced *sodB* transcript levels by 28% in the Tasty Sweet seedlings and 44% in Ambrozja seedlings. The factorial ANOVA test did not confirmed any significant effects of four investigated parameters and their interactions on the transcriptional activity of *sodB* gene in maize seedlings (Table 5). However, the *post-hoc* analysis (Tykey's test; P≤0.05) revealed the significant differences in levels of relative expression of *sodB* gene only between Ambrozja plants infested with 60 *R. padi* aphids (24 hpi) and other experimental variants (Figure 4).

## Discussion

Harmful effects of the aphid colonization on plant growth and development as well as mechanisms underlying various aphid-elicited physiological and biochemical responses within tissues of the host systems have been extensively studied over the last decade [69]–[76]. In Poland, four aphid species have been identified on maize crops: *Metopolophium dirhodum* Walk., *Rhopalosiphum maidis* F., *Rhopalosiphum padi* L. and *Sitobion avenae* F. [77]–[78]. The grain aphid (*S. avenae*) is a monoecious and monophagous hemipteran that colonizes the *Poaceae* plants [79]–[81], whereas the oligophagous *R. padi* migrates between primary hosts (*Prunus* sp.) and a wide spectrum of secondary hosts (cereals and wild grasses) [70], [82]–[84]. The present study is the first to demonstrate differences in the expression profiles of several *sod* genes in maize plants having a susceptible or relatively resistant genotype colonized by monophagous or oligophagous aphids, the salivary secretions of which may differ with respect to activity and toxicity—thereby underlying differences in their ability to induce defense responses in the host. The capacity of host systems to maintain redox balance under aphid attack depends on rapid and efficient functioning of complex cellular antioxidative networks. Deciphering the specific strategies involved in sustaining homeostasis in colonized plants may lead to a better understanding of the resistance mechanisms of plants towards these highly deleterious insects.

Recent studies strongly suggest that ROS play a crucial role in complex plant-insect interactions [85]–[91]. Excessive O_2_^−^ production in plant tissues under adverse conditions may lead to substantial damage to cellular macromolecules (e.g. proteins, lipids, DNA) [27]–[29]. On the other hand, O_2_^−^ can act as a signaling molecule that triggers ROS-dependent defense systems to allow adaptation to stressors. Mai et al. [24] reported that pea aphid (*Acyrthosiphon pisum*) colonization led to significant insect density- and time-dependent enhancement in the rate of O_2_^−^ and H_2_O_2_ production in pea (*Pisum sativum*) seedlings. Moloi and van der Westhuizen [20] also demonstrated that Russian wheat aphid (*Diuraphis noxia*) colonization significantly stimulated H_2_O_2_ accumulation in leaves of the resistant wheat (*T. aestivum,* Tugela line) compared with near-isogenic susceptible plants. These authors postulated that the increased H_2_O_2_ activates signaling pathways that are responsible for the resistance to *D. noxia*. Similarly, Kerchev et al. [22] observed that potato (*Solanum tuberosum*) leaves attacked by green peach aphids (*Myzus persicae*) have nearly twice the H_2_O_2_ than uninfested plants. One of the pivotal functions of H_2_O_2_ in plant tissues is to trigger protein phosphorylation cascades in response to a wide range of environmental stimuli, which leads to widespread induction of stress-related genes [89]–[90]. The DPPH assay has been widely employed to evaluate the total non-enzymatic antioxidative capacity of a diverse array of plant systems. This analytic procedure is based on reduction of an organic DPPH• (1,1-diphenyl-2-picrylhydrazyl) leading to a gradual decline in the absorbance value in parallel with color changes of the reaction mixture from deep violet to pale yellow [92]–[94]. The study demonstrated the variability in the extent of the antioxidative capacity in aphid-colonized seedlings of aphid-tolerant and aphid-susceptible maize cultivars. The relatively resistant cultivar had a markedly stronger deceleration of the scavenging activity of DPPH radicals in response to aphid infestation than the more susceptible variety. Additionally, bird cherry-oat aphid evoked a greater response in the antioxidative activity in maize seedlings than grain aphid, and the changes were proportional to insect abundance and duration of exposure. It should be emphasized that regeneration of the antioxidant pool during a prolonged oxidative burst is progressively less efficient, and therefore the scale of injuries to organelles and cellular compounds is increased under these conditions [25], [31]–[32]. Similar results were obtained by Xie et al. who reported a significant depletion in the DPPH radical scavenging capacity in salt-stressed seedlings of cotton (var. 99B) when compared to the control plants. However, the additional application of coronatine (COR – chlorosis-eliciting phytotoxin) enhanced levels of salt tolerance by acceleration of the antioxidative potential within the treated seedlings [95]. Demiral et al. also evidenced a profound diminution of the antiradical activity in watery extracts of *Olea europaea* (Gemlik cv.) plants subjected to high salinity [96]. It is important to underline that several other researchers confirmed significant modulations in levels of DPPH• scavenging activity in plants exposed to a broad range of environmental stressors, such as chilling [97]–[98], drought [99], high temperature [97], pesticide treatment [100], wounding [101], phytopatogenic viruses, bacteria and fungi [102].

Only a few studies have demonstrated significant aphid-elicited modulation of gene expression in tissues of colonized host plants [91], [103]–[104]. The comparative analyses of *sod* specific expression patterns in the present study revealed that both aphid species tested substantially altered the expression of several *sod* genes in a density- and time-dependent manner. Interestingly, most of the *sod* genes examined were more markedly upregulated in the aphid-relatively resistant Ambrozja plants than in the aphid-susceptible Tasty Sweet plants. It should be noted that both insect species influenced slight fluctuations in expression of *sodB* gene in maize cultivars, however, these modulations were not statistically significant. Kuśnierczyk et al. reported the downregulation of two genes encoding FeSOD (*fsd1* and *fsd2*) in *Arabidopsis thaliana* colonized with cabbage aphids (*Brevicoryne brassicae*) [89]. The same study revealed upregulated expression of four other genes encoding germin-like protein precursors having SOD activity [89]. Kerchev et al. [22] found that the gene encoding a putative cytosolic Cu/ZnSOD was upregulated in *M. persicae*–infested potato plants, whereas the levels of an FeSOD gene transcript gradually declined. Similarly, Dubey et al. demonstrated that the CuSOD 1 gene was upregulated and the FeSOD 3 gene downregulated in leaves of cotton (*Gossypium hirsutum*) infested by cotton aphids (*Aphis gossypii*). These authors postulate that aphids may regulate a similar set of genes to those influenced by plant hormones, microbial infection, or wounding, implying complex crosstalk between the diverse pathways elicited by a broad range of environmental stimuli. Moran et al. [91] also found that the genes encoding cytosolic Cu/ZnSOD1 and a FeSOD were up- and downregulated, respectively, in *A. thaliana* colonized with *M. persicae*. Furthermore, microarray-based analyses of *A. thaliana*–*M. persicae* interactions support the hypothesis that aphid feeding may alter gene expression profiles in a manner similar to wounding or pathogens [91].

In this study, *R. padi* aphids had a greater influence on maize *sod* expression when compared with *S. avenae*. Species-specific differences in the mode of stylet penetration, feeding behaviors, and composition of salivary secretions are likely the main factors determining the scale of aphid-induced injuries and consequent ROS generation in host plants. In support of this model, extensive injuries have been observed in the parenchyma tissue of *R. padi*–infested *T. aestivum* [105], and bird cherry-oat aphid's mouthpart penetration has been shown to disrupt large areas of the mesophyll in leaf tissues of *Prunus padus* [106]. In contrast, stylet insertion and the phloem-feeding style of the grain aphid appear to result in less damage in colonized *T. aestivum* [107].

Taken together, obtained results suggest that the greater SOD response of Ambrozja seedlings relative to Tasty Sweet seedlings might be an important factor in the ability to alleviate the aphid-stimulated oxidative burst and thus may be the basis of Ambrozja's greater ability to survive infestation. Nevertheless, further comprehensive profiling of stress-associated genes in aphid-infested *Z. mays* plants is required to unravel the molecular mechanisms that regulate the highly complex intracellular antioxidant machinery.

## Supporting Information

Table S1 **List of the analysed** ***Z. mays*** **superoxide dismutase (** ***sod*** **) genes quantified using TaqMan® Gene Expression Assays^a)^.** ^a)^ TaqMan® Gene Expression Assays were designed and prepared by Life Technologies (Poland).
(DOC)

Table S2 **A. List of** ***Z. mays*** **genes quantified using Custom TaqMan® Gene Expression Assays^b)^.** ^b)^ Custom TaqMan® Gene Expression Assay were designed by the author and prepared by Life Technologies (Poland)**. B. Sequences of primers designed for amplification of** ***sod9*** **and** ***gapdh*** **genes.** F – forward primer, R – reverse primer. **C. Sequences of TaqMan® fluorescent probes designed for amplification of** ***sod9*** **and** ***gapdh*** **genes.** FAM – 6-carboxyfluorescein, NFQ – 3′–non-fluorescent quencher.
(DOC)
