# Supplementary material for: Differential Expression of Superoxide Dismutase Genes in Aphid-Stressed Maize (Zea mays L.) Seedlings
Source: PLoS One. 2014 Apr 10;9(4):e94847. doi: 10.1371/journal.pone.0094847 (PMC3983269; doi:10.1371/journal.pone.0094847)
Supplement: Table S1 — List of the analysed Z. mays superoxide dismutase ( sod ) genes quantified using TaqMan® Gene Expression Assaysa). a) TaqMan® Gene Expression Assays were designed and prepared by Life Technologies (Poland). (DOC) [file pone.0094847.s001.doc]

**Supporting Information Table S1.**

**List of the analysed *Z. mays* superoxide dismutase (*sod*) genes quantified using TaqMan® Gene Expression Assaysa)**

| **Targeted  *sod* genes** | **GenBank reference sequences** | **Assay identification number** | **Encoded isoenzymes** | **Subcellular localization  of izoenzymes** |
| --- | --- | --- | --- | --- |
| *sod2* | NM_001111865.1 | Zm04058840_g1 | Cu/ZnSOD (SOD2) | Cytosol |
| *sod3.4* | NM_001138523.1 | Zm04077061_s1 | MnSOD (SOD3.4) | Mitochondria |
| *sodB* | NM_001111401.1 | Zm04057396_g1 | FeSOD | Chloroplasts |

^a)^ TaqMan® Gene Expression Assays were designed and prepared by Life Technologies (Poland)
