# Supplementary material for: Differential Expression of Superoxide Dismutase Genes in Aphid-Stressed Maize (Zea mays L.) Seedlings
Source: PLoS One. 2014 Apr 10;9(4):e94847. doi: 10.1371/journal.pone.0094847 (PMC3983269; doi:10.1371/journal.pone.0094847)
Supplement: Table S2 — A. List of Z. mays genes quantified using Custom TaqMan® Gene Expression Assaysb). b) Custom TaqMan® Gene Expression Assay were designed by the author and prepared by Life Technologies (Poland). B. Sequences of primers designed for amplification of sod9 and gapdh genes. F – forward primer, R – reverse primer. C. Sequences of TaqMan® fluorescent probes designed for amplification of sod9 and gapdh genes. FAM – 6-carboxyfluorescein, NFQ – 3′–non-fluorescent quencher. (DOC) [file pone.0094847.s002.doc]

**Supporting Information Table S2.**

**A. List of *Z. mays* genes quantified using Custom TaqMan® Gene Expression Assaysb)**

| **Targeted  genes** | **GenBank reference sequences** | **Assay identification number** | **Encoded isoenzymes** | **Subcellular localization  of izoenzymes** |
| --- | --- | --- | --- | --- |
| *sod9* | NM_001111953.1 | AIWR29R | Cu/ZnSOD (SOD9) | Cytosol |
| *gapdh* | NM_001111943.1 | AIY9ZL7 | GAPDH1 | Cytosol |

^b)^ Custom TaqMan® Gene Expression Assay were designed by the author and prepared by Life Technologies (Poland)

**B. Sequences of primers designed for amplification of *sod9* and *gapdh* genes.**

| **Amplified  gene** | | **Type  of primer** | **Primer sequence** |
| --- | --- | --- | --- |
| *sod9* | | F | CCTGCGAGCAAGGAGCAT |
|  |  | R | TCTGCTCCAGCTGTCACATTTC |
| *gapdh* | | F | AAGCCGGTCACCGTCTTT |
|  |  | R | CATCTTTGCTTGGGGCAGA |

F – forward primer, R – reverse primer.

***C. Sequences of TaqMan® fluorescent probes designed for amplification of sod9 and gapdh genes.***

| **Amplified  gene** | **TaqMan® Probe Sequence** |
| --- | --- |
| *sod9* | FAM-ATGCCGGTGATCTTG-NFQ |
| *gapdh* | FAM-CTTCACTGACAAGGACAAGGCTGCT-NFQ |

FAM – 6-carboxyfluorescein, NFQ – 3’–non-fluorescent quencher.
